# Perfiles analíticos pre-configurados en insuficiencia cardiaca: implementación y uso en el Sistema Nacional de Salud Español

**DOI:** 10.1515/almed-2021-0076

**Published:** 2022-03-07

**Authors:** Luis Almenar Bonet, Ma Teresa Blasco Peiró, Begoña Laiz Marro, Miguel Camafort Babkowski, Antonio Buño Soto, Maria Generosa Crespo-Leiro

**Affiliations:** Unidad de Insuficiencia Cardíaca y Trasplante, Servicio de Cardiología, Hospital Universitario y Politécnico La Fe, Valencia, España; Universidad de Valencia, Valencia, España; Centro de Investigación Biomédica en Red Cardiovascular (CIBERCV), Madrid, España; Unidad de Insuficiencia Cardíaca y Trasplante, Servicio de Cardiología, Hospital Universitario Miguel Servet, Zaragoza, España; Universidad de Zaragoza, Zaragoza, España; Servicio de Análisis Clínicos, Hospital Universitario y Politécnico La Fe, Valencia, España; Servicio de Medicina Interna, ICMiD, Hospital Clínic, Barcelona, España; Universidad de Barcelona, Barcelona, España; Instituto de Investigaciones Biomédicas August Pi i Sunyer (IDIBAPS), Barcelona, España; Servicio de Análisis Clínicos, Hospital Universitario La Paz, Madrid, España; Unidad de Insuficiencia Cardíaca y Transplante Cardíaco, Servicio de Cardiología, Complexo Hospitalario Universitario A Coruña (CHUAC), A Coruña, España; Instituto Investigación Biomédica A Coruña (INIBIC), A Coruña, España; Universidad de A Coruña, A Coruña, España; Centro de Investigación Biomédica en Red de Enfermedades Cardiovasculares (CIBERCV), Madrid, España

**Keywords:** diagnóstico, gestión sanitaria, insuficiencia cardíaca, perfil férrico, perfiles analíticos, seguimiento

## Abstract

**Objetivos:**

El uso de los perfiles analíticos pre-configurados (PAPs) en el contexto de la insuficiencia cardíaca (IC) podría ayudar a realizar un mejor manejo clínico y gestión eficiente del paciente. Los objetivos del estudio son entender el grado actual de implantación de los PAPs en el manejo de la IC en España y conocer la opinión de expertos sobre los mismos, prestando particular atención a los parámetros del metabolismo del hierro.

**Métodos:**

Se recopiló la opinión de expertos en IC en tres fases. FASE 1: nivel de implantación de los PAPs (n=40). FASE 2: ventajas y desventajas de su uso (n=12). FASE 3: grado de conformidad con la composición de tres PAPs específicos de IC (perfil de evaluación inicial, perfil de seguimiento y perfil *de novo*; n=16).

**Resultados:**

Un 62,5% de los hospitales hacen uso de PAPs para el manejo clínico de la IC, sin encontrarse asociación con su nivel de referencia (p=0,132), localización (p=0,486) o presencia de Unidad de Insuficiencia Cardíaca (p=0,737). Los expertos opinaron que emplear los PAPs en la práctica clínica presenta más ventajas que inconvenientes (8 vs. 3), resaltando los beneficios sobre el diagnóstico. Se identificaron un total de 3 motivaciones y 3 barreras para la implantación de los PAPs. Los expertos valoraron positivamente la composición de los 3 PAPs de IC propuestos.

**Conclusiones:**

La estandarización y homogenización de las pruebas de diagnóstico y seguimiento en los pacientes con IC es un área de mejora en los hospitales españoles analizados, a pesar de que los expertos consultados se han mostrado partidarios de su utilización.

## Introducción

En un contexto de aumento del uso de los recursos y el gasto sanitario [1], el uso correcto de los análisis de laboratorio es fundamental para la correcta gestión clínica [2]. Su mala utilización, tanto en exceso como en su falta, debe ser evitada [2]. Existen diversas estrategias para mejorar el uso de los análisis de laboratorio, siendo una de ellas el uso de los perfiles analíticos pre-configurados (PAPs). Estos perfiles, ajustados a las diversas etapas y situaciones asistenciales, podrían ayudar a un mejor control de las enfermedades, optimizando recursos, ahorrando costes, homogeneizando la atención médica y favoreciendo la investigación.

La insuficiencia cardíaca (IC) es un síndrome clínico en el que los pacientes presentan una sintomatología que incluye disnea, hinchazón de tobillos y cansancio, y que suele estar acompañada de diversos signos, como estertores pulmonares, elevación de la presión venosa yugular y edema periférico [3]. Esta condición patológica es consecuencia de una anomalía de la estructura o de la función cardíaca y es considerado un problema de salud pública de primer orden [3]. En los países desarrollados, aproximadamente un 2% de la población adulta padece IC, una prevalencia que aumenta exponencialmente con la edad, siendo inferior al 1% antes de los 50 años y duplicándose posteriormente con cada década, hasta superar el 8% entre los mayores de 75 años [3]. Constituye la primera causa de ingreso hospitalario en personas mayores de 65 años y representa algo más del 2% del gasto sanitario español, suponiendo también una causa frecuente de morbilidad y mortalidad [3], [4], [5], [6]. La IC es una patología de etiología multifactorial que además presenta diversos factores de riesgo [7]. El déficit de hierro es común en pacientes con IC [8], [9], [10] y por ello se recomienda la medición sistemática de los parámetros de hierro (ferritina sérica y saturación de transferrina) en todos los pacientes con sospecha de IC, así como en algunas de las visitas de seguimiento [3]. Sin embargo, es posible que en la práctica clínica habitual estas recomendaciones no se lleven a cabo en su totalidad (lo mismo podría ocurrir en el caso de otros parámetros). Por ello, sería de gran ayuda su inclusión en los PAPs de los pacientes con IC.

La utilización de los PAPs podría ayudar a realizar un mejor control diagnóstico y seguimiento de la IC y de sus comorbilidades. Todo ello supondría un beneficio en la calidad de la actuación asistencial con mejores resultados en salud y una posible disminución de costes tanto directos por pruebas innecesarias realizadas como indirectos por posibles actuaciones tras una prueba mal indicada o incluso por aquellas no realizadas y que se debieran haber solicitado. El objetivo primario del estudio fue conocer la penetración de estos PAPs en las diversas áreas del territorio nacional español, en una patología de alta prevalencia como es la IC. Los objetivos secundarios fueron, conocer las ventajas y barreras actuales que dificultan la implantación de los PAPs, valorar si están contempladas todas las variables necesarias para el control de la enfermedad según las guías de práctica clínica, incluyendo el perfil férrico, y realizar una encuesta para conocer la opinión de expertos en IC sobre los PAPs más útiles.

## Materiales y métodos

### Diseño del estudio y datos recogidos

Durante la planificación metodológica y el trabajo de campo se contó con la participación de Anima Strategic Consulting, una Agencia Médica (AM). Para definir y reclutar los participantes en el estudio se empleó un muestreo intencional no probabilístico buscando la dispersión de la muestra en tipos de centros hospitalarios, localización geográfica y presencia o no de Unidades de Insuficiencia Cardíaca (UIC). Este estudio no ha contemplado el ámbito de la urgencia hospitalaria.

Las entrevistas fueron grabadas tras el consentimiento del participante. En una primera fase (FASE 1), se llevaron a cabo encuestas telefónicas asistidas por ordenador (CATI) a cardiólogos de diversos centros de toda España, durante los meses de noviembre y diciembre de 2019. Durante la FASE 1, se realizó una prospección sobre el grado de automatización de las analíticas en España y el nivel de implementación y uso de los PAPs en el manejo clínico de la IC. Las preguntas de los cuestionarios CATI se diseñaron de forma conjunta con la AM, con respuestas dicotómicas (sí/no). Los participantes de la FASE 1 fueron cardiólogos con más de 5 años de experiencia tras su residencia y con una consulta que tratara, al menos, a 15 pacientes mensuales con IC. Los centros en los que trabajan dichos clínicos y sus principales características pueden verse en la Figura suplementaria 1. El criterio utilizado para la clasificación de los hospitales según su orden de referencia fue obtenido de la Organización Mundial de la Salud [11]. El guion de la entrevista puede consultarse en la Figura suplementaria 2.

Durante la FASE 2, se realizaron entrevistas a expertos en PAPs, en los meses de mayo y junio del año 2020, participando 7 cardiólogos expertos en IC que ya contaban en su hospital con PAPs para el manejo de la IC y 5 jefes de laboratorio clínico (3 de laboratorio interno y 2 de laboratorio externo) que poseían experiencia en la creación de dichos perfiles (Figura suplementaria 3). En esta fase, se adquirió información sobre el proceso de creación de estos PAPs y se identificaron buenas prácticas en su manejo en centros referentes. Debido al contexto COVID-19, las entrevistas, diseñadas y ejecutadas por la AM, se realizaron mediante videollamada. El guion de entrevista se muestra en la Figura suplementaria 4.

El cuestionario CATI de la FASE 1 permitió una recogida de resultados instantánea por parte del entrevistador, mientras realizaba la entrevista. Como control de calidad, cada entrevistador escuchó posteriormente cada entrevista, verificando toda la información. Las entrevistas fueron analizadas individualmente por un investigador distinto al que había realizado la entrevista. Adicionalmente, un cuarto investigador revisó el análisis de 10 entrevistas aleatorias. Tras una reunión conjunta de análisis cros-entrevista y consenso, se obtuvieron los resultados principales del trabajo de campo.

Las entrevistas de la FASE 2 fueron transcritas textualmente. El proceso de análisis fue similar al de la FASE 1, con la diferencia de que el consultor que realizaba la entrevista y el que la transcribía era diferente, por motivos de calidad. Se realizó posteriormente un análisis cualitativo, donde dos investigadores clasificaron las respuestas de los participantes, realizando una puesta en común. Una vez efectuada la clasificación, los mismos investigadores contabilizaron las repeticiones de cada respuesta.

La FASE 3 comenzó con la creación, por parte de los autores, de una propuesta de tres PAPs para el manejo clínico de los pacientes con IC (perfil de evaluación inicial, perfil de seguimiento y perfil *de novo*; Tabla suplementaria 1). Dichos PAPs se construyeron teniendo en cuenta la última evidencia científica y los parámetros más comúnmente analizados en estos tres momentos clave de la enfermedad. Un total de 16 expertos en IC opinaron sobre la composición y utilidad de los mismos, mostrándose a favor o en contra de los parámetros incluidos.

### Análisis estadístico

Las variables cualitativas se expresan mediante su valor absoluto, y las diferencias entre grupos fueron analizadas mediante test Chi-cuadrado o con el test exacto de Fisher. El análisis estadístico se realizó mediante el software GraphPad Prism 9.0 (GraphPad Software, Inc., San Diego, CA).

## Resultados

### Nivel de penetración de los PAPs de pacientes con IC

Todos los hospitales entrevistados contaban con sistemas electrónicos, resultando heterogéneo el proceso de solicitud de las analíticas de los pacientes con IC.

De los 40 hospitales analizados, un 37,5% (15 hospitales) no disponían de PAPs para uso en pacientes con IC, por lo que debían acudir a un sistema de selección individualizada de parámetros (Tabla 1). Por otro lado, el 62,5% de los hospitales (25) contaban con PAPs para el abordaje de la IC. Con un solo clic en el sistema, podían solicitar un conjunto de parámetros determinados para la evaluación del paciente con IC.

**Tabla 1:** Tipos de análisis realizados en los pacientes con IC según tipo de centro hospitalario.

			Pre-configurado	Individualizado	p-Valor
Nivel de referencia	n (%)	1^er^ nivel	3 (7,5)	0	0,132
		2^do^ nivel	8 (20)	9 (22,5)	
		3^er^ nivel	14 (35)	6 (15)	

Localización	n (%)	Comarcal	8 (20)	3 (7,5)	0,486
		Urbano	17 (42,5)	12 (30)	

UIC	n (%)	Sin	8 (20)	6 (15)	0,737
		Con	17 (42,5)	9 (22,5)	

Total	n (%)		25 (62,5)	15 (37,5)	

IC, insuficiencia cardíaca; PAPs, pre-configurado; individualizado, sistema de selección individualizada.

Los resultados del estudio de la FASE 1 demostraron una ausencia de correlación entre el grado de automatización de la solicitud de la analítica en cada centro y el nivel de referencia del hospital (p=0,132; tercer, segundo o primer nivel), su localización (p=0,486; urbano o rural) o la presencia o ausencia de UIC (p=0,737; Tabla 1).

En la Tabla suplementaria 2 se pueden observar los datos referentes a la presencia del perfil férrico (PF) tanto en los PAPs, como en forma de paquetes concretos (en el caso de los hospitales sin PAPs). En este caso, los hospitales de tercer nivel se asocian a la presencia del PF (p=0,048). Sin embargo, ni la localización, ni la presencia de UIC se encuentran relacionadas con la manera de solicitar el análisis del PF en el contexto de los pacientes con IC.

### Ventajas e inconvenientes de los PAPs

**Figura 1:**
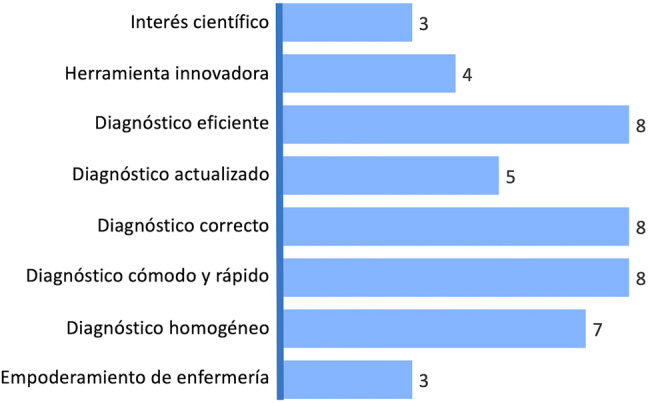
Ventajas de los PAPs. PAPs, perfiles analíticos pre-configurados.

A través de las entrevistas (FASE 2) se pudo comprobar cómo cardiólogos y jefes de laboratorio destacaban grandes ventajas de trabajar con los PAPs (Figura 1). Los motivos por los que ven positivo trabajar con ellos son el interés científico, ser una herramienta innovadora, lograr empoderar a enfermería, así como permitir un diagnóstico eficiente, correcto, actualizado, homogéneo, cómodo y rápido. Destacan como opiniones más citadas las referentes al diagnóstico eficiente, correcto, cómodo y rápido (con 8 votos) y la opinión sobre el diagnóstico homogéneo (7 votos).

**Figura 2:**
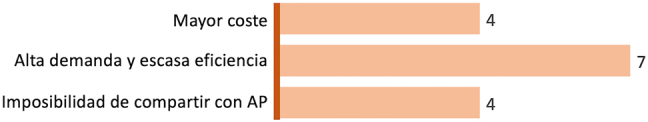
Inconvenientes de los PAPs. AP, atención primaria; PAPs, perfiles analíticos pre-configurados.

Con respecto a los inconvenientes del uso de PAPs, los expertos consultados han indicado como razones el posible mayor coste/eficiencia si no se configuran de forma correcta, la imposibilidad de compartirlo con atención primaria y la alta demanda y escasa eficacia, siendo esta última opción la más repetida (con 7 votos; Figura 2).

### Motivaciones y barreras en el proceso de creación de los PAPs

**Figura 3:**
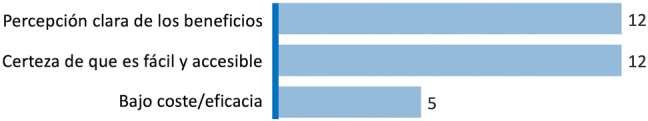
Motivaciones para la creación de los PAPs. PAPs, perfiles analíticos pre-configurados.

En relación a las motivaciones para la creación de los PAPs, los expertos consultados han citado el ratio coste/eficiencia, la percepción clara de los beneficios y la certeza de que es un sistema fácil y accesible (Figura 3). Estas dos últimas contestaciones han sido citadas por unanimidad.

**Figura 4:**
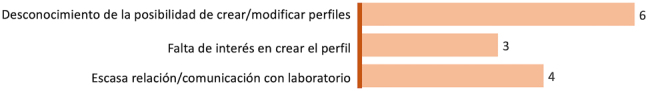
Barreras para la creación de los PAPs. PAPs, perfiles analíticos pre-configurados.

Las opiniones sobre las barreras ante la creación de los PAPs se muestran en la Figura 4. Como se puede apreciar, y por orden creciente del número de repeticiones, los motivos que han contestado son: la falta de interés en la creación, la escasa relación y/o comunicación con laboratorio y el desconocimiento de la posibilidad de crear y modificar los perfiles de análisis.

### Propuesta de PAPs para el manejo clínico de la IC y opinión de los expertos

Los tres PAPs propuestos para el manejo clínico de la IC y su composición pueden verse en la Tabla 2. Tres de los expertos consultados (18.75%), no consideraron oportuna la creación de estos PAPs. Dos de ellos alegaron como motivo haber desarrollado otras alternativas de perfiles, no encontrando necesario crear otros. El otro experto en contra, razonó que su servicio de cardiología tiene un PAP general de cardiología, que considera idóneo para el manejo clínico de los pacientes con IC. Con respecto a la opinión de los 13 expertos (81,25%) que mostraron su conformidad sobre el uso de estos tres PAPs para el manejo clínico de la IC, la gran mayoría se mostró a favor de la composición de los mismos. Con respecto al perfil 1 (perfil de evaluación inicial), los expertos mostraron su unanimidad con respecto a la inclusión de todos los parámetros a excepción de CA-125 (5 en contra) y NT-proBNP (2 en contra). A destacar que la totalidad de los expertos han considerado que se debe medir el perfil férrico dentro de este perfil 1. Un consenso unánime también resultó de la prospección de la composición del perfil 2 (perfil de seguimiento) para la gran parte de los parámetros. En este caso, fueron el ácido úrico y el NT-proBNP los que recibieron dos opiniones en contra de su inclusión. Sobre el perfil 3 (perfil *de novo*), la mayoría de expertos mostraron su conformidad con los parámetros incluidos, aunque sólo alcanzando la unanimidad para el análisis de orina.

**Tabla 2:** Propuesta de PAPs para el manejo clínico de la IC y opinión de los expertos.

PERFIL 1: Perfil de evaluación inicial		
	A favor	En contra
Hemoglobina glicosilada A1c	13	0
Urea	13	0
Glucosa	13	0
Creatinina	13	0
Ácido úrico	13	0
Colesterol total	13	0
Colesterol HDL (directo)	13	0
Colesterol LDL (calculado)	13	0
Triglicéridos	13	0
AST/GOT	13	0
ALT/GPT	13	0
GGT	13	0
Fosfatasa alcalina	13	0
Bilirrubina total	13	0
Proteínas totales	13	0
Albumina	13	0
Iones (Na, K, Cl)	13	0
Ferritina	13	0
Índice de saturación de transferrina (IST)	13	0
CA125	8	5
NT-proBNP	11	2
Tiempo de protrombina	13	0
INR	13	0
Hemograma	13	0

PERFIL 2: Perfil de seguimiento		
	A favor	En contra
Urea	13	0
Glucosa	13	0
Creatinina	13	0
Ácido úrico	11	2
AST/GOT	13	0
ALT/GPT	13	0
GGT	13	0
Fosfatasa alcalina	13	0
Bilirrubina total	13	0
Proteínas totales	13	0
Albumina	13	0
Iones (Na, K, Cl)	13	0
NT-proBNP	11	2
Tiempo de protrombina	13	0
INR	13	0
Hemograma	13	0

PERFIL 3: Perfil de novo		
	A favor	En contra
Hormonas tiroideas (TSH)^a^	11	2
Troponina de alta sensibilidad (T o I)	12	1
PCR (Proteína C Reactiva)	12	1
Metanefrinas en orina^b^	11	2
Orina (Sedimento y anormales, proteinuria)	13	0

IC, insuficiencia cardíaca; PAPs, perfiles analíticos pre-configurados. ^a^T4 libre y T3 libre si TSH alterada. ^b^Valorar su indicación.

## Discusión

La gestión sanitaria se encuentra en la actualidad en un proceso encaminado hacia la digitalización, la automatización y la homogenización de procesos [12]. Por otra parte, el aumento de la carga y del gasto sanitario es un hecho en los países desarrollados y las predicciones muestran que esta tendencia se mantendrá en un futuro próximo [1]. El uso correcto de pruebas analíticas automatizadas y homogeneizadas puede ser una de las soluciones para la disminución de estos costes, así como para un mejor manejo clínico de los pacientes.

Los datos del presente estudio indican claras diferencias en la gestión y uso de las analíticas de laboratorio para el manejo de la IC y la medida de los parámetros del hierro. Por una parte, se ha diferenciado un grupo de hospitales que tienen a su disposición PAPs de IC, constituyendo el 62.5%, frente a un 37,5% que no dispone de los mismos. El estudio muestra también que este hecho no se encuentra asociado al nivel de referencia del hospital, ni a su localización, ni tampoco a la presencia o no de una UIC. También existe disparidad en el modo de solicitar el PF en los hospitales consultados, existiendo en este caso una significación estadística con respecto al nivel de referencia de los mismos. La realidad de la práctica clínica encontrada, contrasta con la opinión de los expertos consultados con respecto a la propuesta de PAPs para el manejo de la IC, donde de manera unánime han opinado que el PF debe ser evaluado. Y es que el déficit de hierro es común en pacientes con IC y se asocia con un menor rendimiento físico, un deterioro de la calidad de vida relacionada con la salud y un mayor riesgo de mortalidad, independientemente de si va acompañado de anemia o no [8], [9], [10, 13]. La guía elaborada por la *European Society of Cardiology* (ESC) publicada en el año 2021 para el diagnóstico y tratamiento de la IC [3], recomienda la medición periódica de los parámetros de hierro en todos los pacientes con IC, así como en las visitas de seguimiento. Conocer el déficit de hierro permite realizar un tratamiento de suplementación a los pacientes, resultando en un beneficio clínico de los mismos [14], [15], [16], [17]. Además de un impacto clínico positivo, el tratamiento con hierro en los pacientes con IC también resulta en un beneficio económico desde el punto de vista de la gestión sanitaria, como así ha demostrado el trabajo de Delgado y colaboradores [18]. Varios estudios, como un meta-análisis llevado a cabo por Khan en 2020 [15], así como dos ensayos clínicos (CONFIRM-HF [17] y AFFIRM-AHF [16]), encontraron que el tratamiento con carboximaltosa de hierro a pacientes con IC reduce el riesgo de hospitalizaciones, lo que presumiblemente también puede asociarse con una disminución en los costes [15], [16], [17]. Por todo ello, la implantación de la medida del PF en el manejo clínico de los pacientes con IC debe estandarizarse, siendo los PAPs una posible herramienta para efectuar este proceso y, de esta manera, lograr en la práctica clínica lo que los expertos consideran idóneo.

Con respecto a las ventajas e inconvenientes del uso de PAPs en el manejo de los pacientes con IC, los expertos consultados añadieron más argumentos a favor que en contra, citando sobre todo conceptos relativos a la mejora en el diagnóstico del paciente. Sobre las motivaciones y barreras para la implementación y uso de los PAPs en el contexto clínico de la IC, se destaca la percepción clara de los beneficios y su facilidad y accesibilidad y en contra el desconocimiento de la posibilidad de la creación y/o modificación de perfiles. A pesar de la percepción a favor del uso de los PAPs y de que los expertos indiquen pocas barreras, tal y como se pone de manifiesto en los resultados de este estudio, la implementación de los PAPs y su uso está lejos de ser mayoritario. Esto puede ser debido, en parte, a la falta de tiempo dedicado a la planificación del que disponen la mayoría de profesionales sanitarios, que impide el desarrollo y la ejecución de nuevos protocolos [19, 20]. Por este motivo, este grupo de autores cree que es importante realizar una concienciación sobre la mejora de este aspecto, que podría ser muy útil para la gestión sanitaria [19, 20].

Como muestran los resultados, la disparidad no sólo se encuentra en el uso o no de PAPs, sino en que el contenido de los parámetros de los mismos también es diverso, lo que imposibilita la comparación de datos entre centros. Todos los expertos consultados mostraron la aceptación de los mismos, aunque con algún comentario sobre alguno de los parámetros a incluir. Cabe resaltar, por ejemplo, las discrepancias con respecto al uso de CA-125 en el primer perfil y es que, a pesar de que existe suficiente evidencia científica al respecto como para considerar su medida como requerimiento para el manejo clínico de la IC [21, 22], su utilización está poco mencionada en las guías clínicas [3]. Aunque menores, también han existido discrepancias en relación a la medida de NT-proBNP, tanto en el perfil 1 como en el 2. En este caso, la medida de este parámetro sí que se encuentra entre las recomendaciones de la guía ESC publicada en 2021 [3]. Sin embargo, los expertos consultados que no lo incluirían en el PAP han indicado que no consideran su medida pertinente para todos los pacientes. Algunos de los expertos consultados en esta FASE 3 (tres en concreto) han comentado también la posible inclusión de algunos de los parámetros del perfil 3 (hormonas tiroideas, troponina cardíaca medida por método de alta sensibilidad, PCR y metanefrinas plasmáticas) dentro del perfil 1, correspondiente a la primera analítica ordinaria. Estos expertos tan solo solicitarían el análisis de la orina (sedimento y anormales, proteinuria) en la analítica de seguimiento en planta de hospitalización. Los tres PAPs son una propuesta de partida, tanto en contenido como en número y el desarrollo de los mismos podría ser abordado en un futuro a través de un proceso de consenso, que podría por lo tanto culminar con unas recomendaciones de uso de PAPs en IC.

Este estudio consta de varias limitaciones. Uno de ellos es el tamaño muestral empleado cuando se compara con el amplio número de profesionales que se encargan del manejo clínico de la IC en España. Es por ello, que la muestra utilizada presenta una amplia representatividad, incluyendo diferentes tipos de hospitales, localizados en diversos territorios y con o sin UIC. Otra de las limitaciones es el uso de CATI para la toma de datos, que podría haberse visto afectado por diversos sesgos, como la ausencia de señales visuales y la pérdida y distorsión de datos [23, 24]. Sin embargo, la utilización de CATI es una forma muy común y aceptada de obtener datos en práctica clínica, investigación en salud pública y epidemiología, ya que permite recopilar una gran cantidad de información e incluso tratar temas delicados [23].

Durante las últimas décadas, varios estudios han descrito esfuerzos para mejorar el uso apropiado de las pruebas de laboratorio [25, 26], tanto en entornos hospitalarios [27, 28], como en atención primaria [29, 30]. Las estrategias para generar el cambio de uso de la utilización de pruebas comprende modificaciones a los sistemas de petición electrónicos, educación dirigida y retroalimentación sobre el comportamiento de las peticiones [31], [32], [33], [34], [35], [36]. Este estudio pretende sumarse a estas estrategias, ofreciendo información sobre el uso clínico real de los PAPs en el contexto de la IC. Uno de los objetivos de la implantación de los PAPs es el de contribuir a la mejora del modelo organizativo y manejo de los pacientes con IC, tal y como recomiendan la Sociedad Española de Cardiología (SEC), la Sociedad Española de Medicina de Urgencias y Emergencias (SEMES) y la Sociedad Española de Medicina Interna (SEMI) [37]. Hasta la fecha, el presente artículo, es el único estudio publicado sobre PAPs en España y muestra que la estandarización y homogenización sobre las pruebas diagnósticas y de seguimiento en los pacientes con IC es todavía una asignatura pendiente. En este trabajo también se incluye una propuesta de PAPs para ser utilizados en la práctica clínica de la IC, así como la valoración de un grupo de expertos al respecto, siendo los resultados positivos. Este trabajo podría constituir un punto de partida para la validación y uso de PAPs en IC, que ayudaría a homogeneizar el seguimiento de los pacientes en todo el sistema nacional de salud, incentivaría la investigación y así mismo podría desencadenar en unos mejores resultados clínicos e incluso en una posible disminución de costes. Son necesarias futuras acciones, que permitan promover, extender e incentivar la implantación y el uso de PAPs en el manejo clínico de la IC.

En conclusión, se puede decir que los PAPs están poco implantados en España. No obstante, la gran mayoría de profesionales sanitarios entrevistados opinan que ayudan a la homogeneización y estandarización de las pruebas diagnósticas de laboratorio en los pacientes con IC, mostrándose partidarios de su implementación y utilización. Sería deseable fomentar una mejor comunicación y coordinación entre los servicios de laboratorio y cardiología, así como mejorar las herramientas tecnológicas, que ayuden a realizar una mejor gestión de las analíticas a través del uso de PAPs en el manejo clínico de la IC.

## Supplementary Material

Supplementary Material

## Material Suplementario

La versión en línea de este artículo incluye material suplementario (https://doi.org/10.1515/almed-2021-0076)
