# Supplementary material for: Perfiles analíticos pre-configurados en insuficiencia cardiaca: implementación y uso en el Sistema Nacional de Salud Español
Source: Adv Lab Med. 2022 Mar 7;3(1):71–8. [Article in Spanish] doi: 10.1515/almed-2021-0076 (PMC10197756; doi:10.1515/almed-2021-0076)
Supplement: Supplementary file 1 — Supplementary Material [file j_almed-2021-0076_suppl.zip › almed-2021-0076_suppl_001.docx]

**Tabla suplementaria 1.**

Perfiles propuestos y momentos y lugar de uso

|  | **Momento de uso** | **Lugar** |
| --- | --- | --- |
| **Perfil 1: Perfil de evaluación inicial** | - Primera analítica solicitada en planta tras ingreso hospitalario - Seguimiento en consulta cada 3-6 meses | - Hospitalización - Ambulatorio |
| **Perfil 2: Perfil de seguimiento** | - Seguimiento en planta de hospitalización cada 2-3 días - Cuando se produce una modificación significativa del tratamiento | - Hospitalización |
| **Perfil 3: Perfil de novo** | - Ante un caso de nuevo diagnóstico, una vez estabilizado el paciente | - Hospitalización - Ambulatorio |
