# Supplementary material for: Perfiles analíticos pre-configurados en insuficiencia cardiaca: implementación y uso en el Sistema Nacional de Salud Español
Source: Adv Lab Med. 2022 Mar 7;3(1):71–8. [Article in Spanish] doi: 10.1515/almed-2021-0076 (PMC10197756; doi:10.1515/almed-2021-0076)
Supplement: Supplementary file 1 — Supplementary Material [file j_almed-2021-0076_suppl.zip › almed-2021-0076_suppl_002.docx]

**Tabla suplementaria 2.**

Tipos de análisis realizados con perfil férrico en los pacientes con IC según el tipo de centro hospitalario.

|  |  |  | **Pre-configurado incluye PF** | **Pre-configurado no incluye PF** | **Individualizado, incluye PF** | **Individualizado, no incluye PF** | **p-valor** |
| --- | --- | --- | --- | --- | --- | --- | --- |
| **Nivel de referencia** | n(%) | 1^er^ nivel | 1 (2,5) | 2 (5) | 0 (0) | 0 (0) | 0,048* |
|  |  | 2^do^ nivel | 4 (10) | 4 (10) | 1 (2,5) | 8 (20) |  |
|  |  | 3^er^ nivel | 12 (30) | 2 (5) | 3 (7,5) | 3 (7,5) |  |
| **Localización** | n(%) | Comarcal | 4 (30) | 4 (10) | 0 (0) | 3 (7,5) | 0,298 |
|  |  | Urbano | 13 (32,5) | 4 (10) | 4 (10) | 8 (20) |  |
| **UIC** | n(%) | Sin | 3 (7,5) | 5 | 1 (2.5) | 5 | 0,132 |
|  |  | Con | 14 (35) | 3 (7,5) | 3 (7.5) | 6 (15) |  |
| Total | n(%) |  | 17 (42,5) | 8 (20) | 4 (10) | 11 (27,5) |  |

Pre-configurado=PAP; Individualizado= sistema de selección individualizada; PF= perfil férrico (Ferritina e Índice de Saturación de Transferrina); *P ≤ 0.05
