# Supplementary figures and images for: Perfiles analíticos pre-configurados en insuficiencia cardiaca: implementación y uso en el Sistema Nacional de Salud Español
Source: Adv Lab Med. 2022 Mar 7;3(1):71–8. [Article in Spanish] doi: 10.1515/almed-2021-0076 (PMC10197756; doi:10.1515/almed-2021-0076)

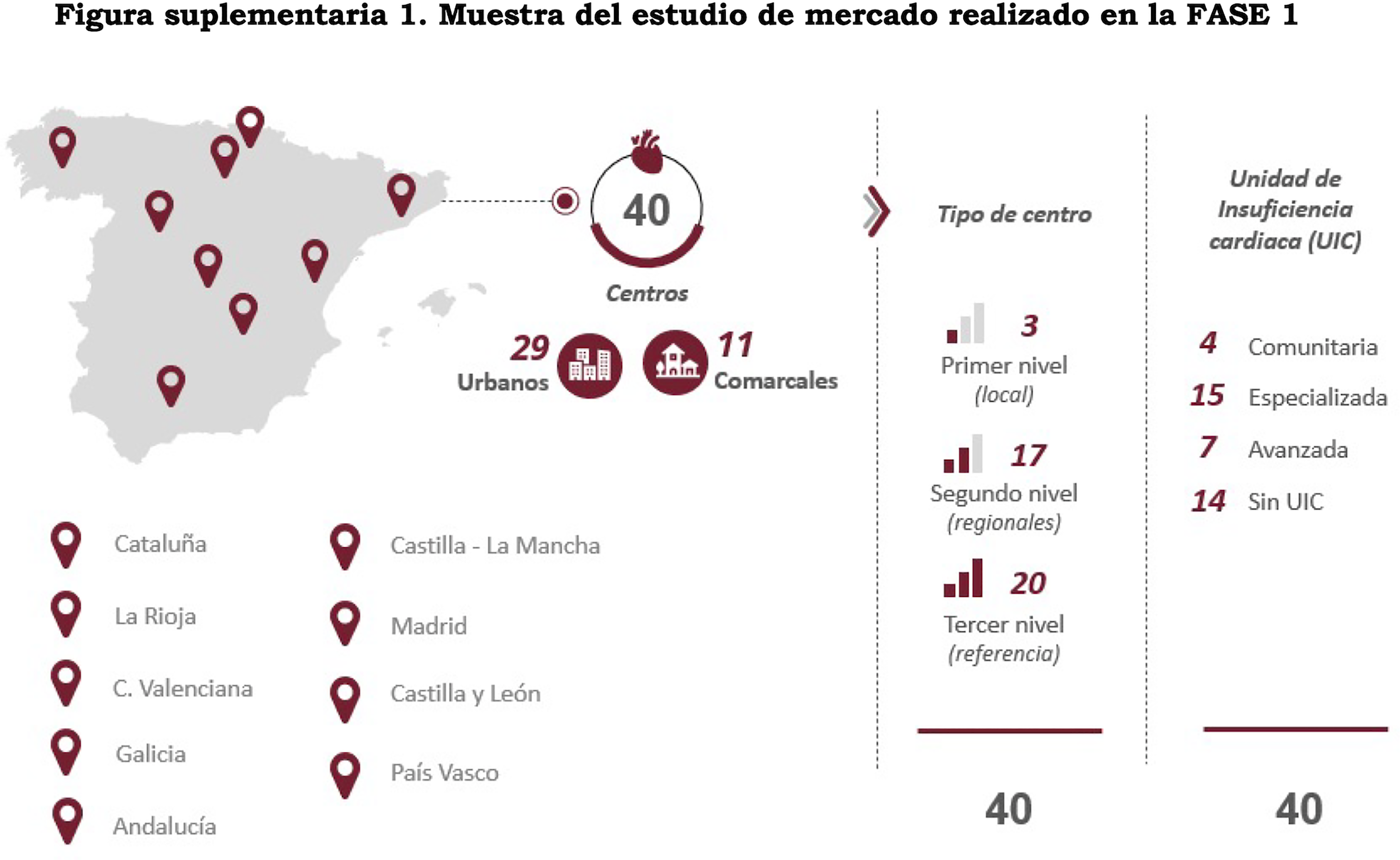

Supplement: Supplementary file 1 — Supplementary Material [file j_almed-2021-0076_suppl.zip › almed-2021-0076_suppl_003.tif]

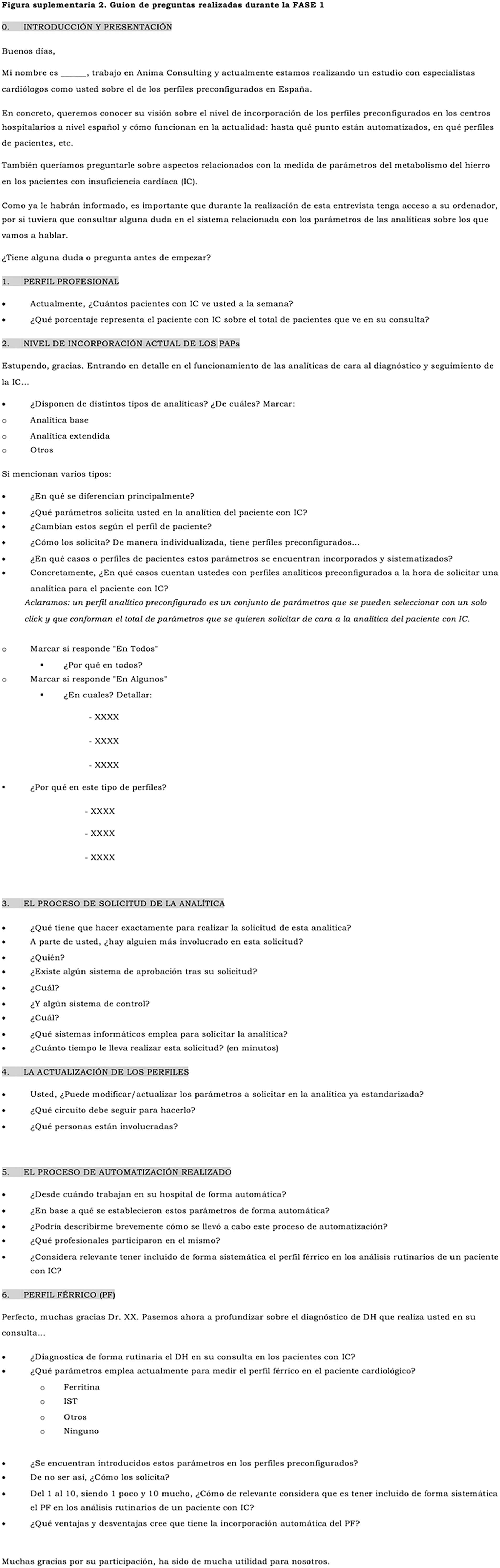

Supplement: Supplementary file 1 — Supplementary Material [file j_almed-2021-0076_suppl.zip › almed-2021-0076_suppl_004.tif]

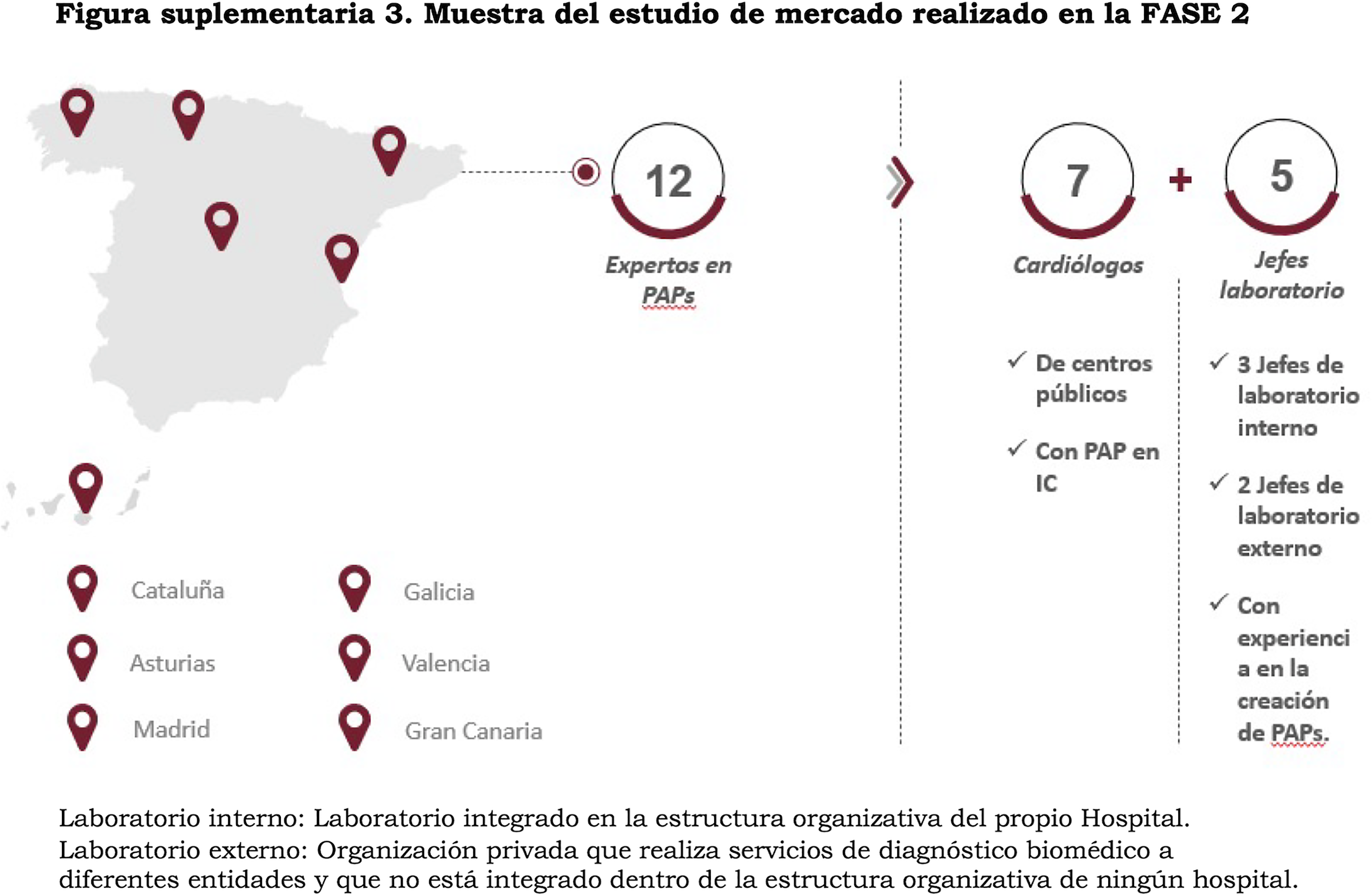

Supplement: Supplementary file 1 — Supplementary Material [file j_almed-2021-0076_suppl.zip › almed-2021-0076_suppl_005.tif]

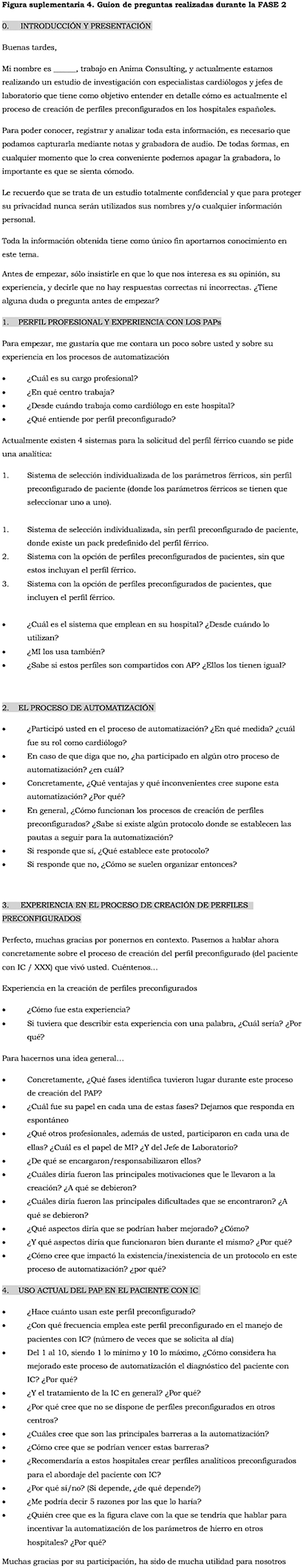

Supplement: Supplementary file 1 — Supplementary Material [file j_almed-2021-0076_suppl.zip › almed-2021-0076_suppl_006.tif]
